# Q fever endocarditis complicating biventricular failure: diagnostic and therapeutic insights from a case report and literature review

**DOI:** 10.3389/fmed.2026.1756873

**Published:** 2026-03-18

**Authors:** Lama Alfehaid, Khawlah Alrubayan, Amal Aleanzi, Monirah Alotaibi, Ahmed Elmubark, Atheer Aldairem, Amira H. Ahmed

**Affiliations:** 1 Department of Pharmacy Practice, College of Pharmacy, King Saud bin Abdulaziz University for Health Sciences, Riyadh, Saudi Arabia; 2 Department of Pharmaceutical Care Services, King Abdulaziz Medical City, Ministry of National Guard-Health Affairs, Riyadh, Saudi Arabia; 3 King Abdullah International Medical Research Center, Riyadh, Saudi Arabia; 4 Adult Cardiology Department, King Abdulaziz Medical City, Ministry of National Guard-Health Affairs, Riyadh, Saudi Arabia

**Keywords:** case reports, *Coxiella burnetii*, culture-negative infections, doxycycline, endocarditis, heart failure, heart valve prosthesis, hydroxychloroquine

## Abstract

**Background:**

Q fever endocarditis (QFE) is a rare, life-threatening infection caused by *Coxiella burnetii*, accounts for 5–6% of culture-negative endocarditis cases and predominantly affects prosthetic or structurally abnormal valves. Diagnosis is frequently delayed by negative blood cultures and subtle or atypical echocardiographic findings.

**Case presentation:**

We describe a 45-year-old man with dual mechanical valves and ischemic cardiomyopathy who presented in cardiogenic shock with acute decompensated heart failure and cardiogenic shock. Transesophageal echocardiography revealed severe prosthetic mitral valve dehiscence with paravalvular regurgitation. Despite broad empiric antimicrobial therapy, blood cultures remained negative until serologic testing confirmed QFE. His clinical course was complicated by severe biventricular failure, recurrent infections, and refractory fluid overload, requiring continuous renal replacement therapy, prolonged doxycycline–hydroxychloroquine therapy, and intensive multidisciplinary care. Surgical intervention was deferred because of prohibitive operative risk, and the patient ultimately died from multiorgan failure.

**Literature review:**

A scoping review of 53 studies, comprising 421 cases on QFE found that patients were predominantly male (77.4%), with a weighted mean age of 53 years. Aortic valve involvement was most frequently reported (57.5% of cases with available valve data), and vegetations were described in 65.1% of cases with reported echocardiographic findings. Blood cultures were negative in 96.2% of cases with available culture data, while Phase I IgG titers ≥1:800 were reported in 83.7% of cases with available serologic data. Doxycycline plus hydroxychloroquine was the most commonly used treatment regimen (52.2%), and cardiac surgery was performed in 58.4% of cases with available surgical data. Reported mortality was 10.6% among cases with available outcome data.

**Conclusion:**

This case highlights the diagnostic challenges of QFE in patients with prosthetic valves and underscores the importance of early serologic testing in the setting of culture-negative endocarditis. The accompanying literature synthesis confirms the central role of serology, the frequent need for surgical intervention, and the persistent challenges in achieving definitive cure.

## Introduction

Q fever endocarditis (QFE) is a rare but clinically significant form of infective endocarditis and an important contributor to blood culture-negative endocarditis. *Coxiella burnetii* accounts for an estimated 5–6% of culture-negative cases (1, 2). Unlike typical endocarditis caused predominantly by gram-positive organisms (3). QFE presents with an indolent course, nonspecific symptoms, and often minimal imaging findings, leading to delayed diagnosis and increased morbidity (4, 5). The clinical spectrum of Q fever varies according to the antigenic phase of *C. burnetii*. Acute infection may be asymptomatic in up to 60% of patients or manifest as a self-limited febrile illness, pneumonia, or hepatitis (4–6). In contrast, chronic Q fever develops in 1–5% of infected individuals and can result in endocarditis, chronic hepatitis, vascular graft infection, mycotic aneurysms, persistent fatigue, osteomyelitis, and other deep-seated infections (4, 7–13). Patients with pre-existing valvular disease, congenital heart lesions, prosthetic valves, malignancy, immunosuppression, or pregnancy are at the highest risk for QFE (9, 10, 12, 14). Diagnosing QFE remains challenging because classic features of infective endocarditis may be absent. Echocardiographic findings are frequently subtle or entirely unremarkable, as vegetations in QFE tend to be small and easily missed even with transesophageal echocardiography, underscoring the importance of serology for diagnosis (2, 8, 15). As a result, phase I and II antibody titers play a pivotal role in diagnostic confirmation, and modifications to the Duke criteria have improved recognition of QFE by incorporating characteristic serologic thresholds (2, 4, 16). Despite these advances, diagnostic delays remain common and are associated with progressive valvular dysfunction, heart failure, and increased mortality (4, 11). Although Q fever is endemic in regions with substantial livestock exposure, including the Middle East, relatively few cases of QFE have been reported in Saudi Arabia, despite frequent contact with camels and consumption of unpasteurized dairy products (5, 6, 17–19). This discrepancy suggests under-recognition and underscores the need for heightened clinical awareness, earlier serologic testing, and improved regional epidemiologic data. This report details a rare and severe case of prosthetic-valve *C. burnetii.* Endocarditis complicated by refractory biventricular heart failure, cardiogenic shock, and multiorgan dysfunction, representing an uncommon phenotype not previously documented in Saudi Arabia. To provide context, we also conducted a narrative review of reported QFE cases from existing literature, analyzing epidemiological features, diagnostic methods, treatment approaches, and outcomes. Both the case and the literature review aim to facilitate earlier detection and improved management of QFE, especially in regions where the disease is often underdiagnosed.

## Methods

### Study design

This manuscript integrates a retrospective single-patient case report with a systematic scoping review of the literature on QFE. The case report was prepared in accordance with the CARE reporting guidelines (20) (CARE checklist provided in the Supplementary materials). The scoping review was conducted and reported in accordance with the Preferred Reporting Items for Systematic Reviews and Meta-Analyses extension for Scoping Reviews (PRISMA-ScR) guidelines. The completed PRISMA-ScR checklist is provided as Supplementary Table S1 (21).

The study protocol was reviewed and approved by the Institutional Review Board of King Abdullah International Medical Research Center (KAIMRC) (Approval number: 00000232525). The requirement for informed consent was waived due to the retrospective and de-identified nature of the data; however, written consent for publication was obtained from the patient’s wife who served as the legally authorized next of kin, given the patient’s critical condition and subsequent death.

### Literature review methodology

We performed a scoping review of published cases of adult QFE. PubMed, Embase, and Cochrane Library were searched from inception to January 2026, using search terms related to Q fever or *C. burnetii*, combined with endocarditis- and valve-related terms. The PubMed strategy included (“Q fever” OR “*Coxiella burnetii*”) AND (endocarditis OR “vascular infection”) AND (valve OR prosthetic). Detailed database-specific search strategies are provided in Supplementary material.

Reference lists of eligible articles were also hand-searched to identify additional relevant reports. Two reviewers independently screened titles and abstracts, followed by full-text assessment of potentially eligible studies. Inclusion criteria comprised English-language case reports, case series, or observational studies describing laboratory-confirmed QFE in adult patients (≥18 years). Data were extracted using a pilot-tested form that captured patient demographics, underlying comorbidities, valve type, diagnostic methods and serologic titers, antimicrobial therapy and duration, surgical interventions, and clinical outcomes. Discrepancies were resolved by consensus.

### Data synthesis and analysis

Given the heterogeneity in study design and reporting across included sources, findings were summarized descriptively. Continuous variables were reported as medians with interquartile ranges or, when available, as means with standard deviations. Categorical variables were summarized as counts and percentages.

For frequently reported outcomes, pooled proportions were calculated using the number of cases with available data as the denominator, acknowledging that denominators varied across variables due to incomplete reporting. No imputation of missing data was performed. Owing to heterogeneity and the descriptive intent of a scoping review, formal meta-analysis was not conducted. Consistent with PRISMA-ScR guidance, study selection is summarized using a PRISMA-ScR flow diagram. Methodological quality of included case reports and case series was assessed using the Joanna Briggs Institute (JBI) critical appraisal checklists as a qualitative assessment of reporting completeness rather than a formal risk-of-bias evaluation, with results summarized in Supplementary Table 1.

### Case report methodology

Patient information was collected through a retrospective review of the institutional electronic health record from admission to discharge. A primary investigator extracted all relevant variables, including demographic data, clinical observations, laboratory and imaging results, hemodynamic assessments, pharmacotherapy records, and procedural reports, using a predefined data-collection template. A second investigator independently re-abstracted 80% of data elements across each domain. Any discrepancies were resolved through review of source documents and, when necessary, consultation with the attending physician of record.

## Case report

A 45-year-old man with a history of ischemic cardiomyopathy and prior dual mechanical aortic and mitral valve replacement with tricuspid repair in 2013 was referred to our tertiary cardiac center on April 30th, 2025, for evaluation of advanced heart failure and potential transplantation. He had experienced multiple recent admissions for recurrent decompensated heart failure. His medical history included chronic atrial fibrillation on warfarin, dyslipidemia, prediabetes, and a remote temporoparietal infarct.

**Figure 1:**
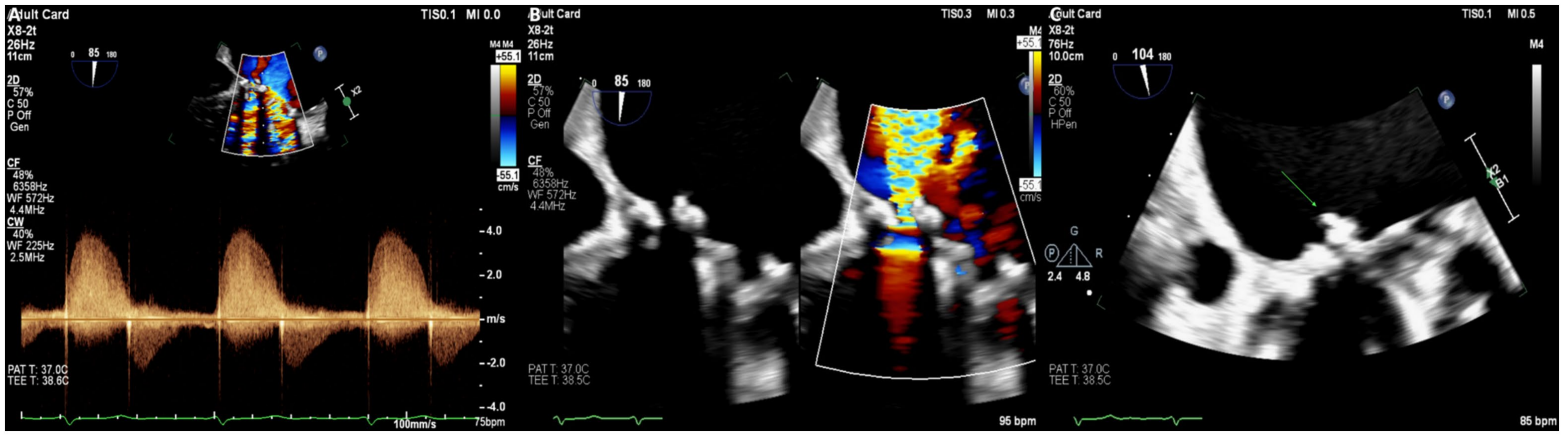
Multimodality echocardiographic assessment demonstrating severe prosthetic mitral valve involvement. **(A)** Continuous-wave Doppler showing dense, holosystolic paravalvular regurgitant flow. **(B)** Color Doppler transesophageal echocardiography revealing severe inferomedial paravalvular leak adjacent to the mechanical mitral prosthesis. **(C)** Transesophageal echocardiography demonstrating a small echogenic mass (arrow) consistent with vegetation at the prosthetic valve annular interface.

At the referring hospital, an initial echocardiogram raised concern for prosthetic mitral valve dysfunction, and broad-spectrum antibiotics were initiated. Upon arrival at our center, the patient was critically ill with hypoxia (SpO₂ 78% on room air), hypotension (systolic blood pressure 87 mmHg), tachypnea, and a depressed level of consciousness. He exhibited features of cardiogenic shock, including severe biventricular dysfunction, volume overload, and end-organ hypoperfusion. However, markedly elevated inflammatory markers, including high procalcitonin and C-reactive protein levels, together with disproportionate vasopressor requirements in the absence of cardiac dysfunction alone, suggested a concurrent distributive component in the setting of active infection. He was therefore assessed as having mixed cardiogenic and distributive shock and was urgently transferred to the Medical Cardiac Intensive Care Unit (MCICU) for vasopressor support, advanced monitoring, and neurological evaluation. Initial laboratory testing demonstrated hyponatremia, renal dysfunction, elevated B-type natriuretic peptide levels, and a subtherapeutic international normalized ratio. Chest radiography showed bilateral pulmonary infiltrates. Transthoracic echocardiography revealed a mechanical mitral valve with a moderate paravalvular leak. Subsequent transesophageal echocardiography demonstrated a severe inferomedial paravalvular dehiscence with pulmonary venous flow reversal and a small mobile echodensity consistent with vegetation (Figure 1). As antimicrobial therapy had been initiated only shortly before transfer, the detected vegetation was considered unlikely to represent a new finding and was instead interpreted as reflecting a pre-existing infectious process.

Shortly after admission, the patient exhibited further mental status deterioration. Brain CT scans revealed no acute ischemia, hemorrhage, or mass effect. Electroencephalography demonstrated severe diffuse slowing, consistent with metabolic or infectious encephalopathy rather than a structural CNS lesion. These findings prompted consultation with the Infectious Diseases service to evaluate culture-negative endocarditis and rule out CNS infection.

Blood cultures repeatedly remained negative. Given the prosthetic valve involvement, persistent shock, encephalopathy, and culture negativity, serologic evaluation for atypical organisms was pursued. On May 22, serology returned positive for *C. burnetii* with elevated Phase I (1,64) and Phase II (1,512) IgG titers, confirming Q fever prosthetic valve endocarditis. The patient was started on doxycycline and hydroxychloroquine to be continued for at least 12 months.

**Figure 2:**
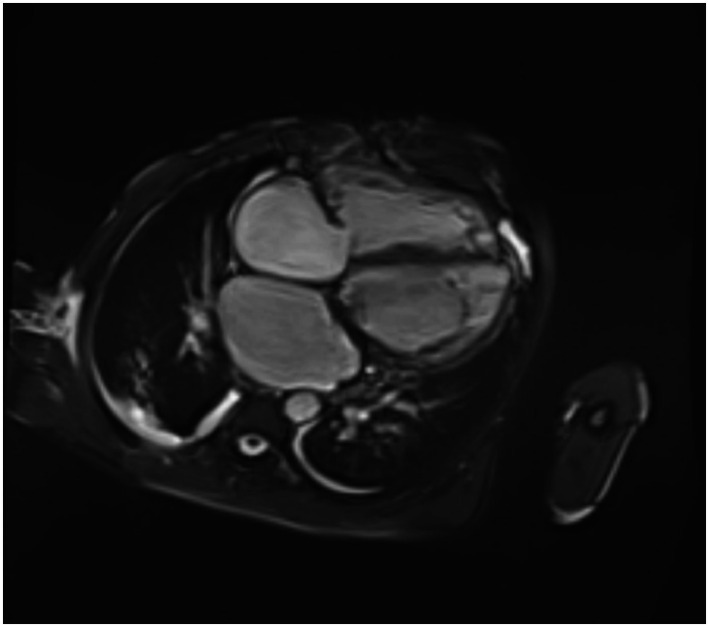
Cardiac MRI (short-axis cine sequence) showing severe biventricular dilatation and markedly reduced systolic function, with disproportionate right ventricular involvement, reflecting advanced biventricular failure in the context of prosthetic valve endocarditis.

Despite targeted therapy, he developed progressive biventricular failure. Cardiac MRI demonstrated a severely dilated left ventricle (EF 39%) and severely reduced right ventricular ejection fraction (12%) (Figure 2), while right heart catheterization revealed markedly elevated filling pressures and increased pulmonary vascular resistance. Continuous renal replacement therapy (CRRT) was initiated for refractory congestion and renal dysfunction.

Cardiac surgery was consulted on two occasions; however, operative intervention was deemed prohibitively high risk due to severe biventricular dysfunction, active infection, and ongoing hemodynamic instability.

On June 12, the patient experienced an asystole/pulseless electrical activity cardiac arrest, with return of spontaneous circulation. Neurological recovery remained poor, and prolonged mechanical ventilation via tracheostomy was required. His MCICU course was further complicated by recurrent hospital-acquired and ventilator-associated pneumonias, refractory ascites related to severe tricuspid regurgitation, and multidermatomal herpes zoster. During episodes of pneumonia, respiratory cultures grew *Stenotrophomonas maltophilia* (heavy growth on multiple occasions) and *Escherichia coli* (scanty growth on one occasion), and antimicrobial therapy was managed according to institutional protocols for nosocomial infections.

Cardiac computed tomography performed on July 16 demonstrated normally functioning mechanical aortic and mitral prostheses without evidence of abscess, pseudoaneurysm, or perforation, confirming that the severe regurgitation originated from paravalvular dehiscence rather than intrinsic prosthetic valve dysfunction.

On July 24, the patient developed septic shock, with blood cultures subsequently growing *Candida auris*. He was treated with liposomal amphotericin B. In early August, placement of a percutaneous gastrostomy tube was complicated by pneumoperitoneum and suspected peritonitis, which was managed non-operatively. His condition continued to decline with worsening hemodynamics and recurrent tense ascites.

By late August, he developed disseminated intravascular coagulation, refractory metabolic acidosis, and progressive multiorgan failure. The patient died on August 31, 2025.

This case highlights the diagnostic challenges of culture-negative prosthetic valve endocarditis, particularly when presenting with undifferentiated shock and encephalopathy, and underscores the importance of early serologic testing for *C. burnetii*, especially in patients with prosthetic valves in whom Q fever may otherwise be under-recognized. Severe biventricular failure and prohibitive surgical risk were key contributors to the fatal outcome.

To contextualize this patient’s presentation and outcomes, we summarize the findings of published QFE cases.

## Literature search and case identification

**Figure 3:**
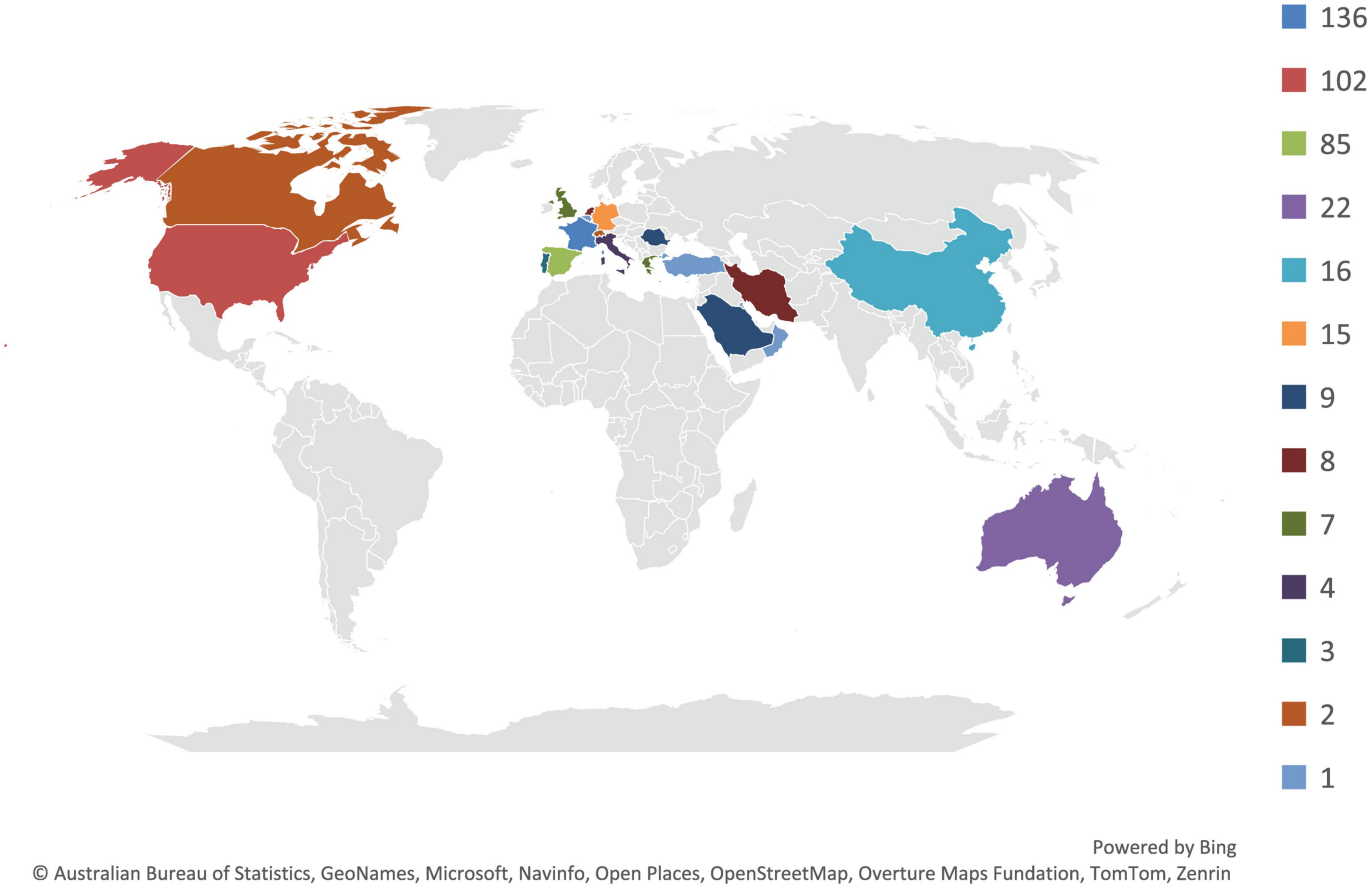
Global distribution of reported Q fever endocarditis cases included in the updated scoping review (*n* = 421). Countries are color-coded according to the number of reported cases, with darker shades representing higher case counts. Legend categories correspond to the following case ranges: ≥134, 97, 85, 22, 15, 9, 8, 7, 4, 3, 2, and 1 case(s). Countries with no reported cases are shown in gray.

The database search yielded 636 records. After removing duplicates and screening titles and abstracts, 53 studies (case reports and case series) met the predefined inclusion criteria and were included in the scoping review (22–68), comprising 421 adult patients with laboratory-confirmed QFE. The global distribution of reported cases is shown in Figure 3. Study selection is summarized in the PRISMA-ScR flow diagram (Supplementary Figure S1).

### Patient characteristics

Across the 53 included studies, 421 cases of QFE were identified. Patients were predominantly male (326/421, 77.4%), with 64/421 (15.2%) female and 31/421 (7.4%) cases not reporting sex. The weighted mean age was 53.0 years.

Underlying cardiovascular disease was common. Among cases with available data, valvular heart disease was reported in nearly half of patients, including both native and prosthetic valve pathology. Mechanical prosthetic valves were frequently represented, underscoring their role as a major predisposing factor for chronic infection. Additional reported risk factors included congenital heart disease, immunocompromised status, and prior infective endocarditis. Animal or livestock exposure was inconsistently reported, and a substantial proportion of cases occurred without an identifiable zoonotic exposure. Because reporting across studies was heterogeneous, denominators varied by variable; all pooled proportions were calculated using the number of cases with available data for each characteristic. The key demographic, clinical, microbiological, management, and outcome data of the included cases are summarized in Table 1.

**Table 1:** Characteristics and summary of the included cases (*n* = 421).

Study (Author, Year)	Country	Cases (*n*)	Mean/Median Age	Male (%)	VHD (%)	Mechanical Valve (%)	Surgery (%)	Cure (%)	Mortality (%)
Jang, 2018 (22)	Korea	8	54	87.5	NR	100	NR	37.5	NR
Habedank, 2024 (23)	Germany	1	55	100	0	0	0	100	0
Panou, 2007 (24)	Greece	1	55	100	0	0	NR	0	0
Yaghmaie, 2015 (25)	Iran	1	72	0	100	100	100	100	0
Allan-Blitz, 2018 (26)	USA	1	38	100	0	0	0	100	0
Mogollón, 2011 (27)	Spain	83	44	78.3	50.6	16.9	73.5	66.3	13.3
Cotar, 2011 (28)	Romania	9	61	77.8	NR	NR	NR	NR	NR
Wang, 2024 (29)	China	1	43	100	0	100	NR	NR	0
Deyell, 2006 (30)	Canada	1	31	100	100	100	100	0	0
Alabdely, 2020 (31)	Saudi Arabia	1	22	0	100	0	100	100	0
Rafailidis, 2006 (32)	Greece	1	65	100	100	100	100	100	0
Karakousis, 2006 (33)	USA	1	31	100	100	100	0	0	100
Kampschreur, 2013 (34)	Netherlands	3	74	66.7	100	0	100	0	0
Kokkini, 2008 (35)	Greece	5	53	100	80	40	40	100	0
McCaul, 1994 (36)	Australia	3	33–40	100	100	100	100	100	0
Oteo, 2012 (37)	Spain	2	51–74	100	0	0	100	50	0
Eldin, (70)	France	100	65	79	40	36	12	NR	NR
Mühlemann, 1995 (38)	Switzerland	2	55.5	100	NR	NR	100	100	0
Morgans, 1969 (39)	UK	1	40	100	100	100	0	0	100
Angelakis, 2014 (40)	Saudi Arabia	2	29	100	100	NR	50	NR	0
Sonsöz, 2020 (41)	Turkey	1	35	100	100	0	100	100	0
Elzein, 2019 (42)	Saudi Arabia	19	19	89.5	100	5.3	26.3	NR	10.5
Han, 2017 (43)	China	6	50	100	100	16.7	33.3	83.3	0
Raizada, 2016 (44)	USA	1	39	0	100	0	100	100	0
Zhang, 2022 (45)	China	8	49	87.5	75	12.5	50	75	12.5
Elgouhari, 2016 (46)	USA	1	57	100	100	0	0	0	0
Million, 2016 (47)	France	9	53	77.8	0	0	0	66.7	11.1
Straily, 2017 (53)	USA	93	57	82.8	48.4	NR	NR	NR	5.4
Varma, 1980 (54)	N. Ireland	8	43	62.5	100	75	25	75	12.5
Kremer, 2018 (55)	Germany	1	33	100	0	0	100	NR	0
Loyen, 2024 (56)	Germany	1	62	100	100	0	100	100	0
Grey, 2023 (57)	Australia	1	68	100	100	0	0	NR	0
Alqallaf, 2020 (58)	Kuwait	1	43	100	100	100	0	0	0
Moreira Marques, 2021 (59)	Portugal	1	79	100	100	0	0	0	100
Mohammed, 2024 (60)	USA	1	66	0	0	0	0	0	0
Santos, 2025 (61)	Portugal	1	79	0	100	100	0	0	0
Bozza, 2023 (62)	Italy	1	55	100	100	100	0	0	0
Afrasiabian, 2024 (63)	Iran	1	67	0	100	0	100	0	0
Shah, 2015 (64)	USA	1	52	0	100	100	100	0	0
King, 2025 (65)	USA	1	20	100	0	100	100	0	0
Van Noten, 2022	Belgium	1	43	100	100	100	0	0	0
Al Suqri, 2024 (67)	Oman	1	34	100	100	0	100	100	0

VHD, valvular heart disease. Percentages are calculated within individual studies. NR indicates data not reported in the original publication.

Percentages reflect proportions within individual studies and are not pooled estimates.

### Clinical presentations

Most cases were classified as chronic Q fever (412/421, 97.9%), consistent with *C. burnetii’s* known predilection for indolent endocardial infection. Clinical manifestations were variable and often nonspecific.

Among cases with reported symptoms, fever ≥38 °C was documented in 204/308 (66.2%), while fatigue was reported in 87/161 (54.0%). Splenomegaly and hepatomegaly were observed in 94/260 (36.2%) and 74/256 (28.9%), respectively. Anemia was a frequent laboratory abnormality, reported in 106 of 162 (65.4%) cases with available data. Afebrile presentations were also described, highlighting the diagnostic challenge of QFE.

### Valve involvement and echocardiographic findings

Valve involvement was predominantly left-sided. Among cases with reported valve localization, aortic valve involvement was most frequent (131/228, 57.5%), followed by the mitral valve (76/228, 33.3%). Right-sided involvement was less common, with tricuspid valve involvement in 11/130 (8.5%) and pulmonary valve involvement in 25/145 (17.2%). Multivalvular disease was reported in a subset of cases.

Echocardiographic findings were heterogeneous. Vegetations were reported in a substantial proportion of cases with available imaging data, while abscess formation, prosthetic valve dehiscence, and new or worsening valvular regurgitation were less consistently documented. These findings reflect the often subtle and atypical echocardiographic features of QFE, particularly in prosthetic valve settings.

### Diagnostic approaches

Diagnostic confirmation relied predominantly on serologic testing. Blood cultures were negative in 304/316 cases (96.2%), emphasizing the limited utility of conventional microbiological cultures in QFE.

Among cases with reported serology, Phase I IgG titers ≥1:800 by indirect immunofluorescence assay were positive in 261/312 (83.7%), consistent with chronic infection. Phase II IgG and IgM antibodies were variably reported. Molecular diagnostics, including PCR on blood or excised valve tissue, were described in a limited number of cases and were typically adjunctive rather than primary diagnostic modalities.

### Management strategies

Antimicrobial therapy varied across studies but was generally prolonged. The most commonly reported regimen was doxycycline plus hydroxychloroquine, used in 165/316 cases (52.2%) with available treatment data. Doxycycline monotherapy was administered in 52/318 cases (16.4%), while alternative regimens, including fluoroquinolone-based or combination therapies, were less frequently reported.

The duration of antimicrobial therapy, when specified, was typically long-term, most often extending beyond 12 months, in line with guideline recommendations for chronic QFE.

### Surgical intervention and clinical outcomes

Cardiac surgery was performed in 185/317 cases (58.4%) with available procedural data, most commonly for prosthetic valve involvement, severe valvular dysfunction, or complications such as abscess formation.

**Figure 4:**
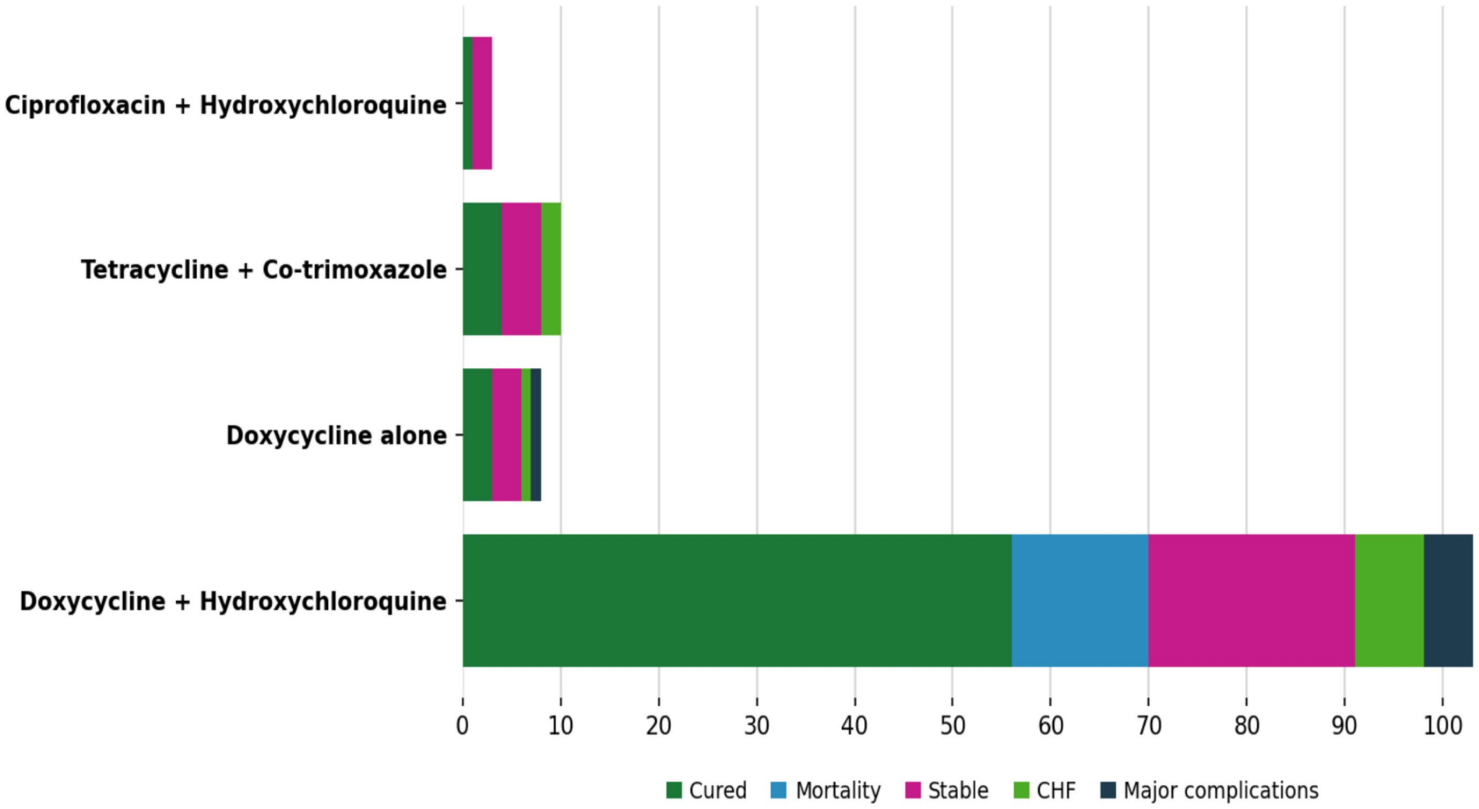
Clinical outcomes according to antimicrobial treatment regimens in QFE. Distribution of clinical outcomes according to antimicrobial treatment regimens among reported cases of Q fever endocarditis. Outcomes include cure, mortality, stable disease, heart failure progression, and major complications. Percentages are calculated among cases with available outcome data for each treatment regimen.

Clinical outcomes were variably reported, as shown in Figure 4. Among cases with available outcome data, mortality occurred in 41/387 patients (10.6%). Cure, as defined by the original reports (clinical stability with serologic improvement or resolution), was documented in 146/262 cases (55.7%). Other reported outcomes included stable disease, progression to heart failure, or survival with significant complications.

## Discussion

QFE is a diagnostically challenging entity with heterogeneous and often subtle clinical manifestations, particularly in patients with prosthetic valves or pre-existing structural heart disease. The present case illustrates an unusually aggressive phenotype characterized by prosthetic mitral valve dehiscence, severe paravalvular regurgitation, and profound biventricular failure. While QFE most commonly affects left-sided valves, extensive right ventricular dysfunction and combined biventricular involvement, as observed in this patient, are rarely reported and represent a severe end of the disease spectrum (4, 5).

Initial transthoracic echocardiography in this case suggested only moderate paravalvular leakage; however, subsequent transesophageal imaging revealed a more extensive inferomedial prosthetic valve dehiscence with a small vegetation. This diagnostic delay is consistent with prior observations that vegetations in QFE are typically small, atypical, and easily missed, particularly in prosthetic valve settings, underscoring the importance of high clinical suspicion and advanced imaging modalities when QFE is suspected (68). Cardiac magnetic resonance imaging and invasive hemodynamic assessment further demonstrated marked right ventricular dysfunction and elevated filling pressures, highlighting the value of multimodality evaluation in complex presentations.

Definitive diagnosis relied on serologic testing, with elevated Phase I and II IgG titers supporting chronic QFE despite values below the classic ≥1:800 Phase I threshold. This finding is consistent with documented variability in serologic responses, particularly in patients who have received empirical antibiotics or who present early in the disease course (69). These nuances reinforce the need to interpret serology within the broader clinical context rather than relying solely on rigid cutoff values.

### Comparison with published literature

The clinical features observed in this case align with patterns identified in our updated scoping review of 421 reported QFE cases, in which aortic valve involvement predominated and prosthetic valves represented a major predisposing factor. In contrast, significant right-sided involvement or severe biventricular failure, central features of this case, were infrequently documented, suggesting that QFE may manifest more aggressively in individuals with complex structural heart disease or long-standing prosthetic valves (4, 5).

Consistent with the literature, our patient’s blood cultures were negative, reflecting the well-established limitations of conventional microbiological techniques in QFE. In the pooled analysis, blood culture negativity was reported in approximately 96% of cases with available data, emphasizing the central role of serology in diagnosis and the importance of early testing in patients with culture-negative endocarditis (1–3). Across published reports, 58–60% of patients underwent cardiac surgery, with improved outcomes generally observed in those who were hemodynamically stable and referred early for operative intervention.

In contrast, our patient’s clinical course was dominated by refractory shock, progressive biventricular failure, and multiorgan dysfunction, rendering surgical intervention prohibitive. This outcome is consistent with prior reports indicating markedly worse prognosis among QFE patients who present with advanced heart failure or persistent hemodynamic instability, in whom surgical risk often outweighs potential benefit (4).

In our systematic review, overall mortality was 10.6%. This suggests that our patient’s clinical course represents an extreme of the disease spectrum. The disproportionate right ventricular involvement (12% vs. 39%) is particularly notable, as isolated or predominant right heart failure is rarely emphasized in QFE literature. We hypothesize that this may have resulted from septal microabscesses identified on cardiac MRI causing conduction abnormalities and mechanical dysfunction, severe tricuspid regurgitation exacerbating RV volume overload; and/or pulmonary hypertension secondary to chronic left-sided heart failure and mitral regurgitation.

Despite prompt initiation of doxycycline and hydroxychloroquine, the recommended regimen for chronic QFE involving prosthetic valves (3, 5), the patient’s condition deteriorated due to recurrent nosocomial infections, renal failure requiring renal replacement therapy, *Candida auris* septicemia, and ultimately died. These overlapping complications highlight the substantial therapeutic challenges encountered when QFE occurs in the setting of advanced structural heart disease and critical illness, particularly when definitive surgical management is not feasible.

Several limitations should be acknowledged. First, the accompanying scoping review is predominantly based on case reports and small case series, which limits the ability to account for study-level heterogeneity or to perform formal meta-analytic comparisons. Pooled proportions and weighted means were therefore used for descriptive purposes only and should be interpreted with caution, particularly in the presence of missing or inconsistently reported data. In addition, the single-patient nature of the case limits generalizability. Nevertheless, key strengths include an updated and comprehensive literature review, transparent handling of missing data, and detailed multimodality phenotyping of a rare and severe clinical presentation. Together, these elements provide valuable insight into an uncommon but clinically important manifestation of QFE and highlight areas where earlier recognition and intervention may influence outcomes.

## Conclusion

QFE remains a diagnostic challenge, particularly in patients with prosthetic valves or pre-existing structural heart disease. This case illustrates how culture-negative presentation, subtle imaging findings, and early empiric antibiotic exposure can delay recognition of *C. burnetii* infection. The patient’s course, which included severe mitral paravalvular dehiscence, profound biventricular failure, refractory shock, and multiorgan dysfunction, highlights a severe phenotype rarely described in literature. Findings from our scoping review reinforce that serologic testing is essential for timely diagnosis, that surgical intervention plays a central role when feasible, and that outcomes are significantly worse among hemodynamically unstable patients. Early consideration of Q fever in culture-negative endocarditis, especially in endemic regions or in patients with prosthetic valves, may improve clinical decision-making and patient outcomes. Further studies are needed to better characterize high-risk phenotypes and optimize management strategies for QFE in critically ill populations.

## Data Availability

The original contributions presented in the study are included in the article/Supplementary material, further inquiries can be directed to the corresponding author.
