# Supplementary material for: Q fever endocarditis complicating biventricular failure: diagnostic and therapeutic insights from a case report and literature review
Source: Front Med (Lausanne). 2026 Mar 18;13:1756873. doi: 10.3389/fmed.2026.1756873 (PMC13038586; doi:10.3389/fmed.2026.1756873)
Supplement: Supplementary file 1 [file Table_1.docx]

# CARE Checklist

| Checklist Item | Reported Information |
| --- | --- |
| Title | Diagnosis + 'case report' wording included. |
| Keywords | Keywords include disease + 'case report'. |
| Abstract – Introduction | Unique aspects described. |
| Abstract – Symptoms/Findings | Main findings are described. |
| Abstract – Diagnosis/Interventions/Outcomes | Provided. |
| Abstract – Conclusion | Clear takeaway. |
| Introduction | Summarizes rarity, significance, and literature. |
| Patient Information – De-identified Data | Provided. |
| Patient Information – Primary Concerns | Provided. |
| Patient Information – Medical/Psychosocial History | Medical history provided; psychosocial not relevant. |
| Past Interventions | Provided. |
| Clinical Findings | Clinical exams and investigations are described. |
| Timeline | Narrative timeline included. |
| Diagnostic Assessment – Tests | All diagnostics included. |
| Diagnostic Challenges | Culture-negative presentation and subtle imaging. |
| Diagnosis | Q fever endocarditis confirmed by serology. |
| Prognosis | Poor due to severe biventricular failure. |
| Therapeutic Intervention – Type | Antibiotics, ICU care, CRRT, and antifungals. |
| Therapy Administration | Durations are described when applicable. |
| Changes in Therapy | Escalation of support is described. |
| Follow‑up – Outcomes | Disease progression and complications are described. |
| Follow‑up – Additional Tests | Serial imaging included. |
| Adherence/Tolerability | No issues reported. |
| Adverse Events | Candida auris sepsis, zoster, etc. |
| Discussion – Strengths & Limitations | Included. |
| Discussion – Literature Review | Comprehensive review included. |
| Discussion – Rationale for Conclusions | Provided. |
| Conclusion | Clear takeaway message included. |
| Patient Perspective | Not reported (patient deceased). |
| Informed Consent | IRB waiver documented. |
