# Supplementary material for: Q fever endocarditis complicating biventricular failure: diagnostic and therapeutic insights from a case report and literature review
Source: Front Med (Lausanne). 2026 Mar 18;13:1756873. doi: 10.3389/fmed.2026.1756873 (PMC13038586; doi:10.3389/fmed.2026.1756873)
Supplement: Supplementary file 2 [file Table_2.docx]

**Supplementary Materials**

**Search Strategies**

**PubMed (MEDLINE)**

The PubMed database was searched using a combination of MeSH terms and free-text keywords as follows:

(

"Q Fever"[MeSH Terms]

OR "Q fever"[Title/Abstract]

OR "Coxiella burnetii"[MeSH Terms]

OR "Coxiella burnetii"[Title/Abstract]

)

AND

(

"Endocarditis"[MeSH Terms]

OR endocarditis[Title/Abstract]

OR "vascular infection"[Title/Abstract]

)

AND

(

valve[Title/Abstract]

OR prosthetic[Title/Abstract]

OR "prosthetic valve"[Title/Abstract]

)

AND

(

"Case Reports"[Publication Type]

OR "case series"[Title/Abstract]

OR "Observational Study"[Publication Type]

)

AND

(

"Adult"[MeSH Terms]

OR adults[Title/Abstract]

**Cochrane Library**

**The Cochrane Library was searched using the following strategy:**

("Q fever" OR "Coxiella burnetii")

AND

(endocarditis OR "vascular infection")

AND

(valve OR prosthetic OR "prosthetic valve")

**Embase**

**The Embase database was searched using Emtree terms and free-text keywords:**

('q fever'/exp OR 'q fever':ti,ab

OR 'coxiella burnetii'/exp OR 'coxiella burnetii':ti,ab)

AND

('endocarditis'/exp OR endocarditis:ti,ab

OR 'vascular infection':ti,ab)

AND

(valve:ti,ab OR prosthetic:ti,ab

OR 'prosthetic valve':ti,ab)

AND

('case report'/exp OR 'case series':ti,ab

OR 'observational study'/exp)

AND

('adult'/exp OR adults:ti,ab)

**Supplementary Table 1.** Methodological quality appraisal of included Q fever endocarditis case reports and case series using Joanna Briggs Institute (JBI) checklists

| **Domain** | **Case Reports (n = 36)** | **Case Series (n = 17)** |
| --- | --- | --- |
| Clear patient demographics described | 36 (100%) | 17 (100%) |
| Clear patient history and timeline | 36 (100%) | 17 (100%) |
| Diagnostic methods clearly reported | 36 (100%) | 17 (100%) |
| Intervention(s) or treatment(s) clearly described | 36 (100%) | 14 (82.4%) |
| Clinical outcomes clearly reported | 36 (100%) | 17 (100%) |
| Adverse events or complications reported | 23 (63.9%) | 11 (64.7%) |
| Adequate follow-up period | 30 (83.3%) | 15 (88.2%) |
| Overall methodological quality (≥70% items fulfilled) | 36 (100%) | 16 (94.1%) |

Methodological quality was assessed using Joanna Briggs Institute (JBI) critical appraisal checklists for case reports and case series. Percentages are calculated using the number of included studies within each category. The assessment was qualitative and descriptive, reflecting the predominance of case-based evidence.

**Identification of studies via databases and registers**

Records removed *before screening*:

Duplicate records removed (n = 182)

Records identified from databases (n = 636)

PubMed (n = 218)

Embase (n = 289)

Cochrane Library (n = 129)

Records identified from registers (n = 0)

**Identification**

Records screened

(n =440)

Records excluded**

(n =364)

Reports sought for retrieval

(n =76)

Reports not retrieved

(n = 3 )

**Screening**

Reports excluded (n = 20):

No individual case-level data (n = 8)

Duplicate or overlapping cohorts (n = 6)

Chronic Q fever without endocarditis (n = 4)

Insufficient diagnostic confirmation (n = 2)

Reports assessed for eligibility

(n =73)

Studies included in scoping review (n = 53)

Case reports (n = 36)

Case series (n = 17)

**Included**

**Figure S1.** PRISMA-ScR flow diagram summarizing the identification, screening, and inclusion of studies on Q fever endocarditis.
